# Supplementary material for: Model-based characterization of the equilibrium dynamics of transcription initiation and promoter-proximal pausing in human cells
Source: Nucleic Acids Res. 2023 Oct 27;51(21):e106. doi: 10.1093/nar/gkad843 (PMC10681744; doi:10.1093/nar/gkad843)
Supplement: gkad843_Supplemental_File [file gkad843_supplemental_file.pdf]

Supplementary Information for: Model-based characterization of the equilibrium dynamics of transcription initiation and promoter-proximal pausing in human cells

Yixin Zhao[1], Lingjie Liu[1,2], Rebecca Hassett[1], and Adam Siepel[1,2,*]

[1]Simons Center for Quantitative Biology, Cold Spring Harbor Laboratory, Cold Spring Harbor, NY
[2]Graduate Program in Genetics, Stony Brook University, Stony Brook, NY
[*]Corresponding author: asiepel@cshl.edu

Supplementary Tables

symbol	parameter
α	Potential initiation rate (relative)
β	Pause escape rate (relative)
γ	Termination rate (relative)
ζ_i	Per nucleotide elongation rate
λ	Scaling factor representing read depth
χ	Compound parameter representing the read-depth-scaled ratio of the initiation rate to the elongation rate
φ	Landing-pad occupancy
ω	Effective initiation rate (relative)
f_k	Fraction of cells with pause site k
k	Position of the pause site
L	Length of the pause peak
N	Total length of the gene
s	Sum of all read counts within a gene
s_p	RNAP center-to-center spacing
t	Sum of Y_k
X_k	Number of read counts at position k
Y_k	Number of read counts that derives from the cells has pause site at k

Supplementary Table S1: Summary of parameters used in the model

symbol	parameter	values
α	Potential initiation rate (relative)	$\alpha\zeta$ = 0.1, 1, 10 events/min.
β	Pause escape rate (relative)	$\beta\zeta$ = 0.1, 1, 10 events/min.
ζ	Elongation rate	Truncated Normal Distribution with<br>Mean = 2,000 bp/min.<br>SD = 1,000 bp/min.<br>Max = 2,500 bp/min.<br>Min = 1,500 bp/min.
k	Position of the pause site (fixed)	50 bp downstream of TSS
	Position of the pause site (variable)	Truncated Normal Distribution with<br>Mean = 50 bp<br>SD = 25 bp<br>Max = 17 bp<br>Min = 200 bp
s_p	RNAP center-to-center spacing	33, 50, 70 bp
Δt	Time slice	10^{-4} min.
T	Total time	40 min.
C	Total cells simulated	20,000 cells
N	Total length of the simulated gene	2,000 bp in simulation<br>20,000 bp in Poisson sampling

Supplementary Table S2: Summary of parameters used in simulation

Supplementary Figures

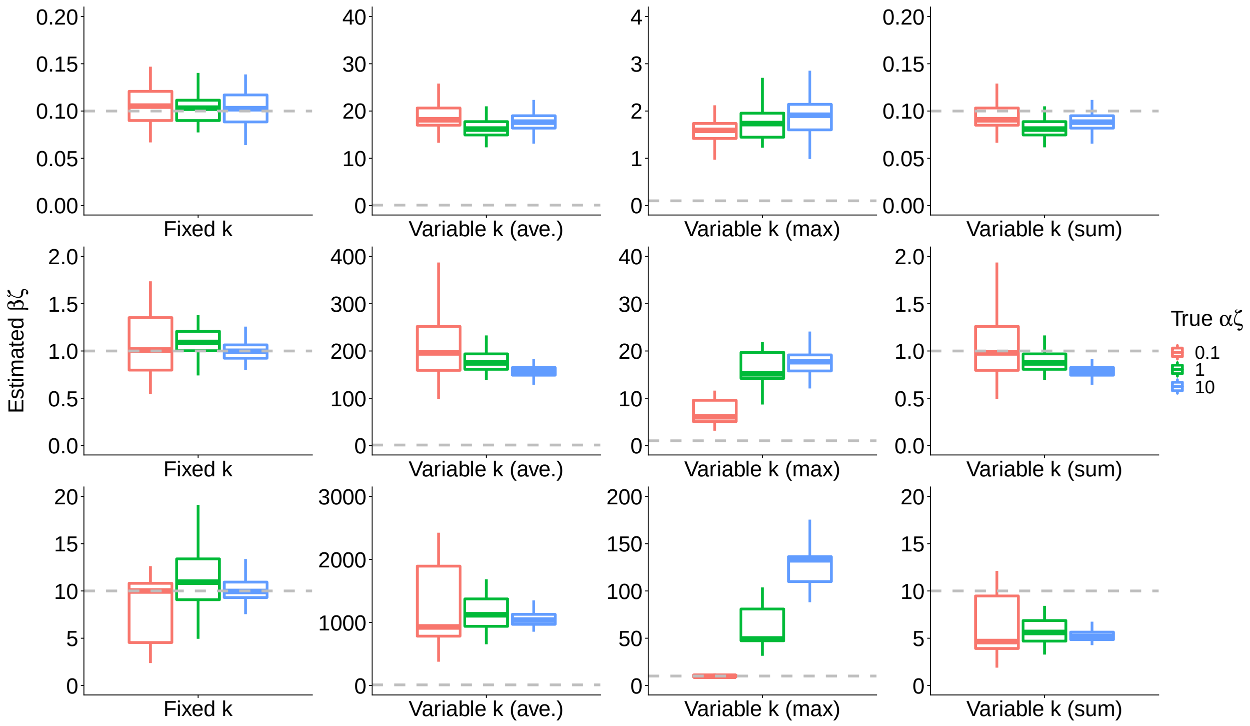

Supplementary Figure S1: Estimated values of $\beta\zeta$ under the initial model for simulated true values of $\beta\zeta \in \{0.1, 1.0, 10\}$ (top to bottom) and $\alpha\zeta \in \{0.1, 1, 10\}$ (see key), when the pause-site k is fixed (first column) or variable across cells (second to last column) in simulation. This is an expanded version of **Fig. 3B** showing a broader range of $\beta\zeta$ values. In the second column, β is estimated using the average read-depth in the pause peak, while in the third and last column, it is estimated using the maximum or sum of read counts. As in that case, dashed lines indicate the ground truth; boxplots summarize 50 replicates of the simulation; box boundaries indicate 1st and 3rd quartiles, and horizontal line indicates median. A value of $\zeta = 2$ kb/min is assumed so that $\alpha\zeta$ and $\beta\zeta$ can be assumed to have units of events per minute. Pause sites occur at a mean position of $k = 50$ nt. In the variable case, we assume a Gaussian distribution with a standard deviation of 25 nt.

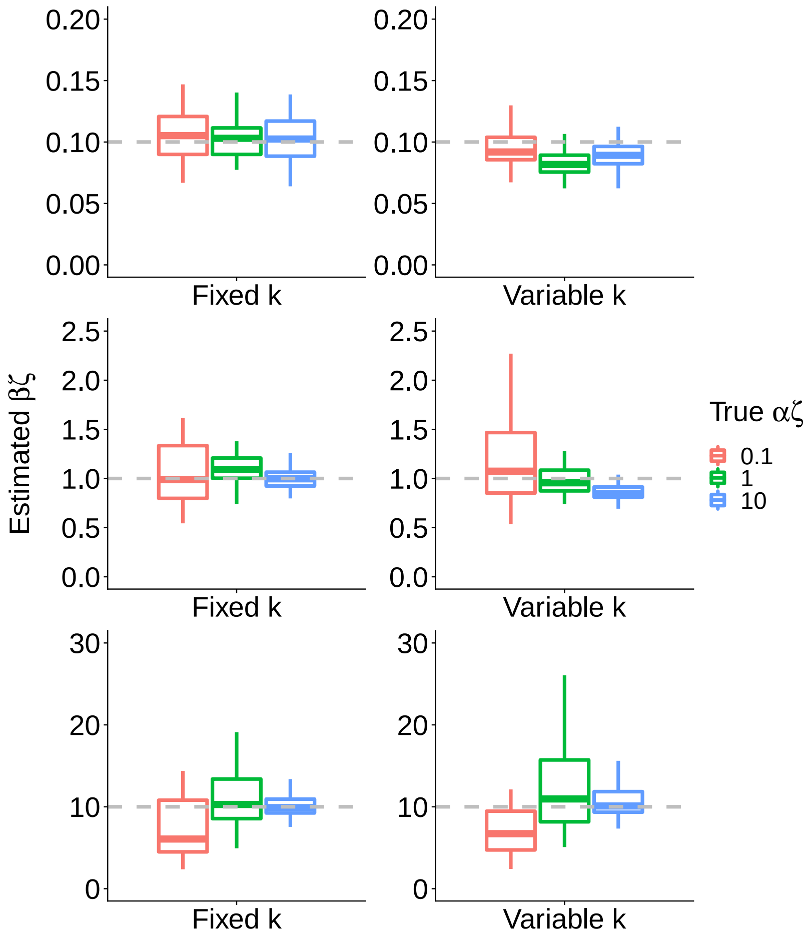

Supplementary Figure S2: Estimated values of $\beta\zeta$ under the variable-pause-site model for simulated true values of $\beta\zeta \in \{0.1, 1.0, 10\}$ (top to bottom) and $\alpha\zeta \in \{0.1, 1, 10\}$ (see key), when the pause-site k is fixed (left) or variable across cells (right) in simulation. This is an expanded version of **Fig. 4A** showing a broader range of $\beta\zeta$ values. As in that case, dashed lines indicate the ground truth; boxplots summarize 50 replicates of the simulation; box boundaries indicate 1st and 3rd quartiles, and horizontal line indicates median. A value of $\zeta = 2$ kb/min is assumed so that $\alpha\zeta$ and $\beta\zeta$ can be assumed to have units of events per minute. Pause sites occur at a mean position of $k = 50$ nt. In the variable case, we assume a Gaussian distribution with a standard deviation of 25 nt.

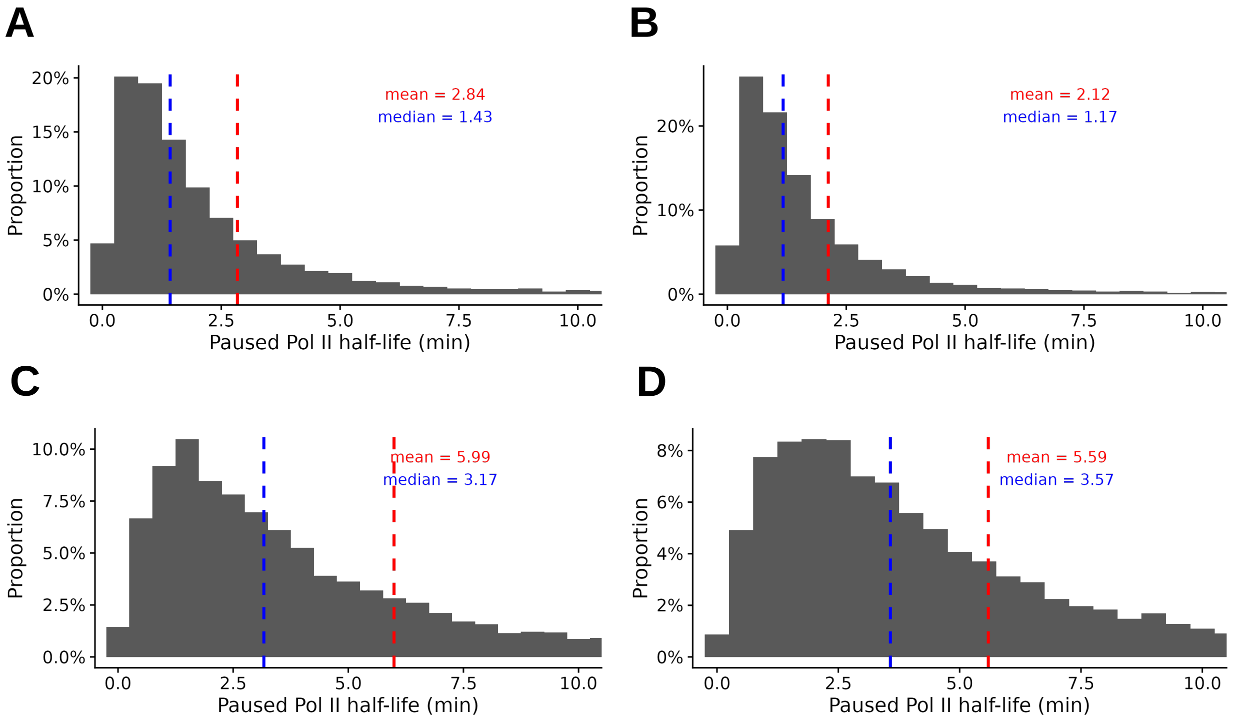

Supplementary Figure S3: Distributions of half-lives for paused RNAPs for **(A)** the untreated K562 cells from Vihervaara et al. [1], **(B)** the untreated K562 cells from Dukler et al. [2], **(C)** the heat-shock-treated K562 cells from Vihervaara et al. [1], and **(D)** the celastrol-treated (160-min) K562 cells from Dukler et al. [2]. The histograms are truncated at 10 min. for clarity. See **Materials & Methods** for details on the calculation of half-lives from β estimates.

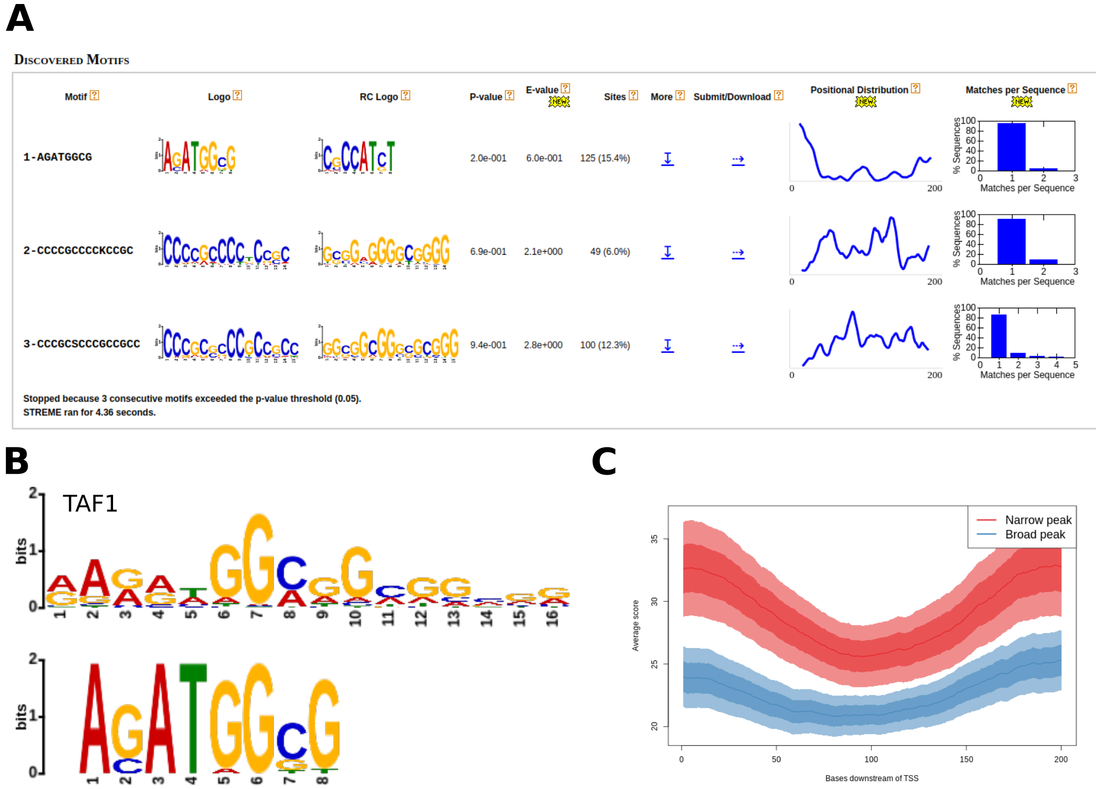

Supplementary Figure S4: Motifs identified in pause peaks from the control sample from the heat-shock data set [1] **A.** Motifs enriched in the 10% genes with narrowest pause peaks (smallest $\hat{\sigma}^2$) compared with the 10% of genes with broadest peaks (largest $\hat{\sigma}^2$) as identified by STREME [3]. **B.** The top candidate from STREME matches the binding motif of TAF1 [4]. **C.** TAF1 ChIP-seq signals from ENCODE [5] for genes with narrow (red) and broad (blue) pause peaks downstream of the TSS.

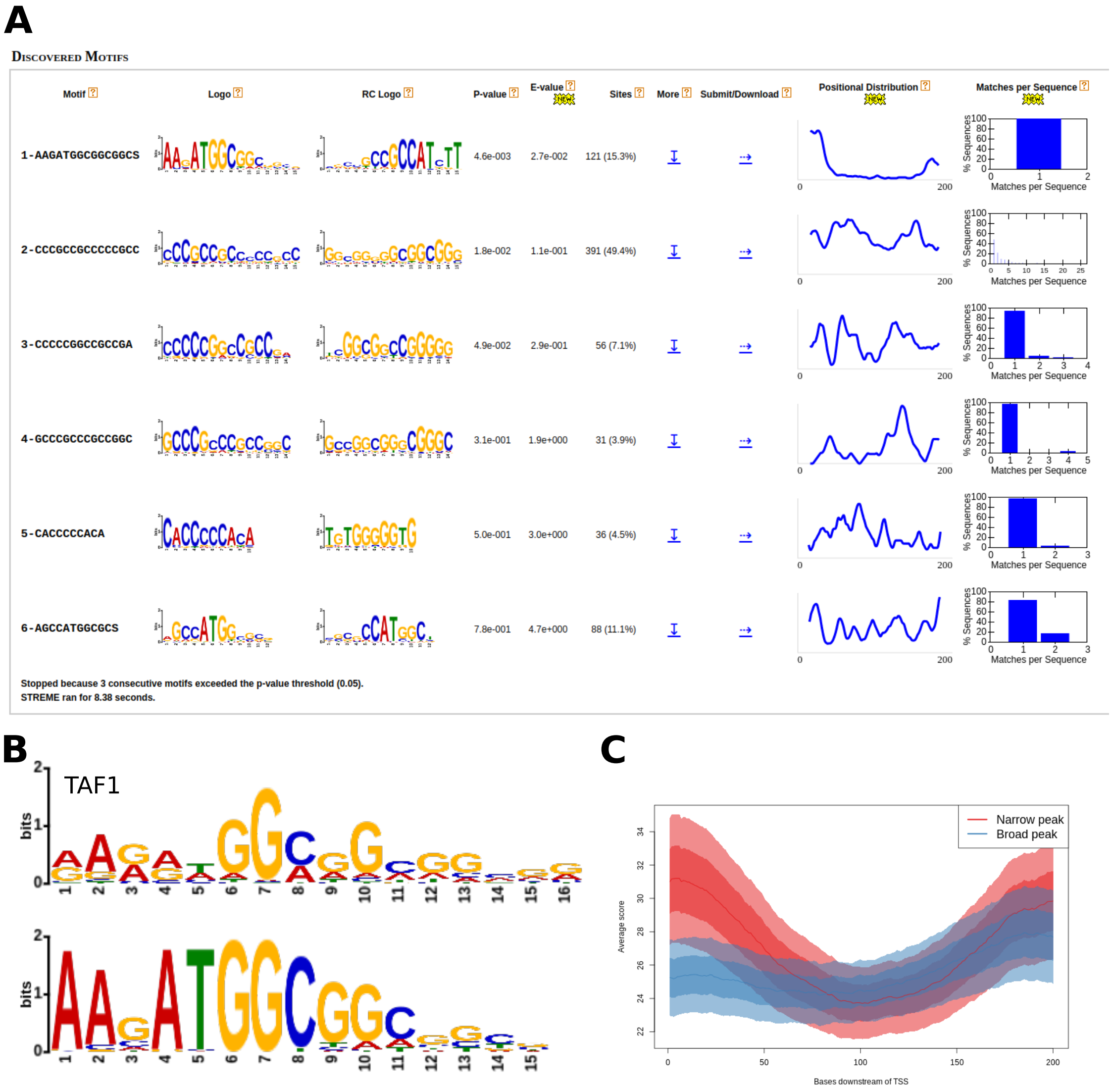

Supplementary Figure S5: Motifs identified in pause peaks from control sample from the celastrol data set [2] **A.** Motifs enriched in the 10% genes with narrowest pause peaks (smallest $\hat{\sigma}^2$) compared with the 10% of genes with broadest peaks (largest $\hat{\sigma}^2$) as identified by STREME [3]. **B.** The top candidate from STREME matches the binding motif of TAF1 [4]. **C.** TAF1 ChIP-seq signals from ENCODE [5] for genes with narrow (red) and broad (blue) pause peaks downstream of the TSS.

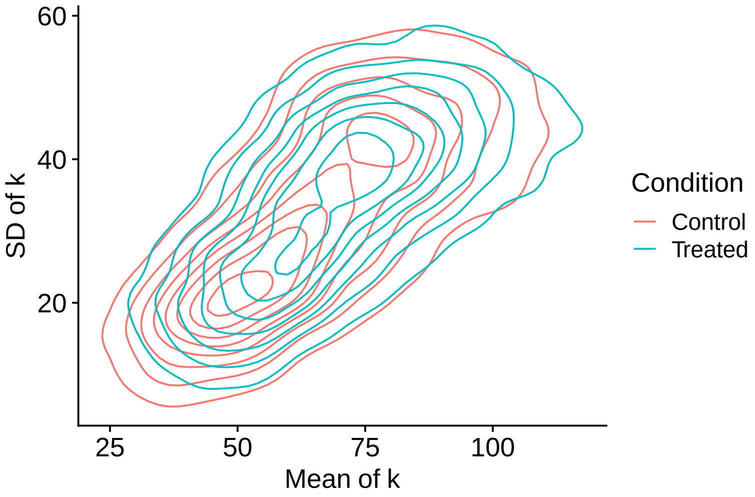

Supplementary Figure S6: Contour plot showing the distribution of estimated means (horizontal axis) and standard deviations (vertical axis) of the pause peak position k, under the Celastrol ("Treated") and Control conditions. Data from ref. [2]. Compare with **Fig. 4E**.

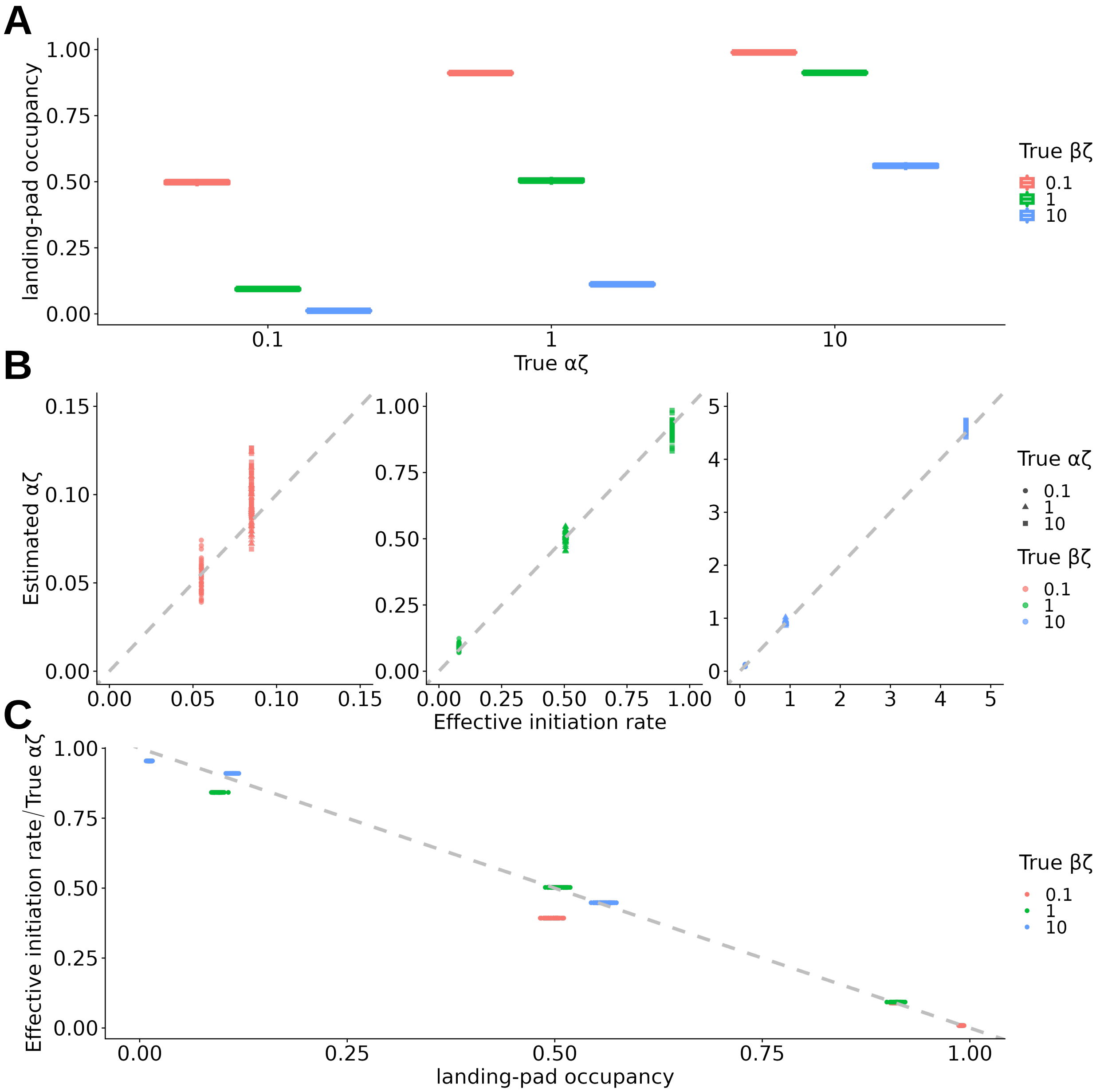

Supplementary Figure S7: Auxiliary data from simulations revealing prevalance of steric hindrance. **A.** Fraction of cells in which the "landing pad" (here, the first 50 nt) for a potential new initiation event is already occupied by an RNAP, for $\alpha\zeta \in \{0.1, 1, 10\}$ (left to right) and $\beta\zeta \in \{0.1, 1, 10\}$ (see key). **B.** Rates at which initiation rates successfully occur ("effective initiation rate") vs. estimates of $\alpha\zeta$, for true $\alpha\zeta \in \{0.1, 1, 10\}$ (left to right) and $\beta\zeta \in \{0.1, 1, 10\}$ (see key). The estimates of $\alpha\zeta$ are much closer to the effective initiation rates than to the true initiation rates. **C.** Landing-pad occupancy (as in panel **A**) vs. "correctness" of estimated $\alpha\zeta$, as measured by the ratio of the estimated value to the true value. The correctness decreases approximately linearly with the landing-pad occupancy.

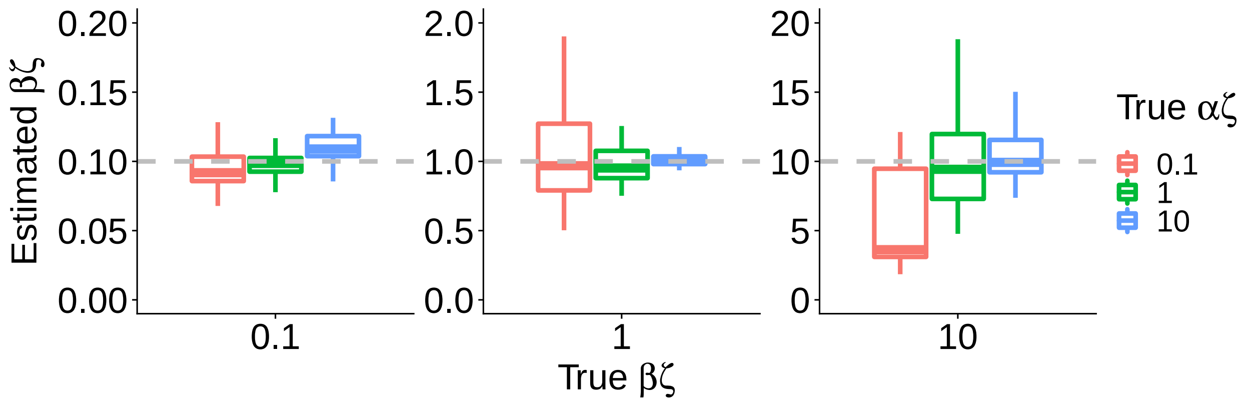

Supplementary Figure S8: Estimated values of $\beta\zeta$ under the steric hindrance model for simulated true values of $\beta\zeta \in \{0.1, 1.0, 10\}$ (left to right) and $\alpha\zeta \in \{0.1, 1, 10\}$ (see key). Estimates are close to the truth, except for the case of $\beta\zeta = 10$, $\alpha\zeta = 0.1$, for which the pause peak was poorly defined. As in previous plots, dashed lines indicate the ground truth; boxplots summarize 50 replicates of the simulation; box boundaries indicate 1st and 3rd quartiles, and horizontal line indicates median. A value of $\zeta = 2$ kb/min is assumed so that $\alpha\zeta$ and $\beta\zeta$ can be assumed to have units of events per minute. Pause sites are variable with a mean position of $k = 50$ nt and a standard deviation of 25 nt.

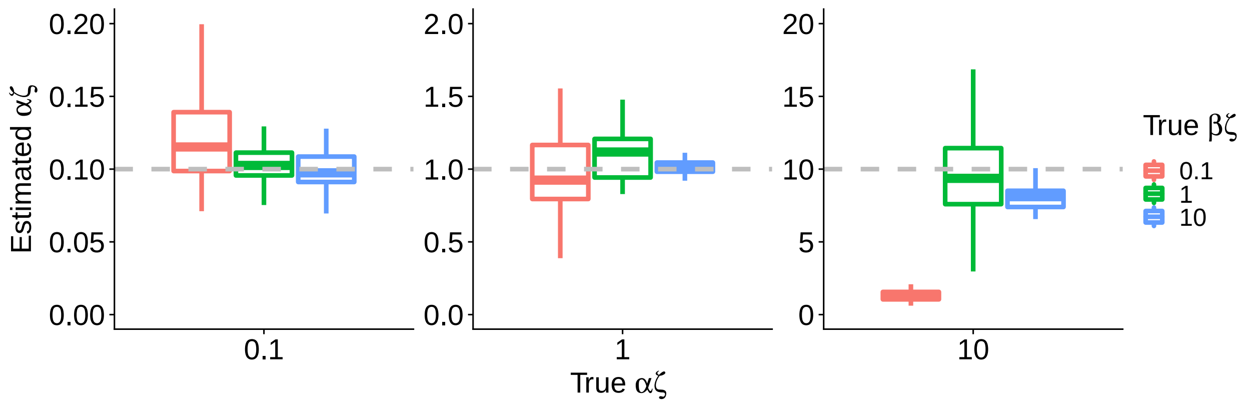

Supplementary Figure S9: Estimated values of $\alpha\zeta$ under the steric hindrance model, for $\alpha\zeta \in \{0.1, 1, 10\}$ (left to right) and $\beta\zeta \in \{0.1, 1, 10\}$ (see key). The poor estimate for $\alpha\zeta = 10, \beta\zeta = 0.1$ reflects under-estimation of ϕ near the boundary of $\phi = 1$. As in previous plots, dashed lines indicate the ground truth; boxplots summarize 50 replicates of the simulation; box boundaries indicate 1st and 3rd quartiles, and horizontal line indicates median. A value of $\zeta = 2$ kb/min is assumed so that $\alpha\zeta$ and $\beta\zeta$ can be assumed to have units of events per minute. Pause sites are variable with a mean position of $k = 50$ nt and a standard deviation of 25 nt.

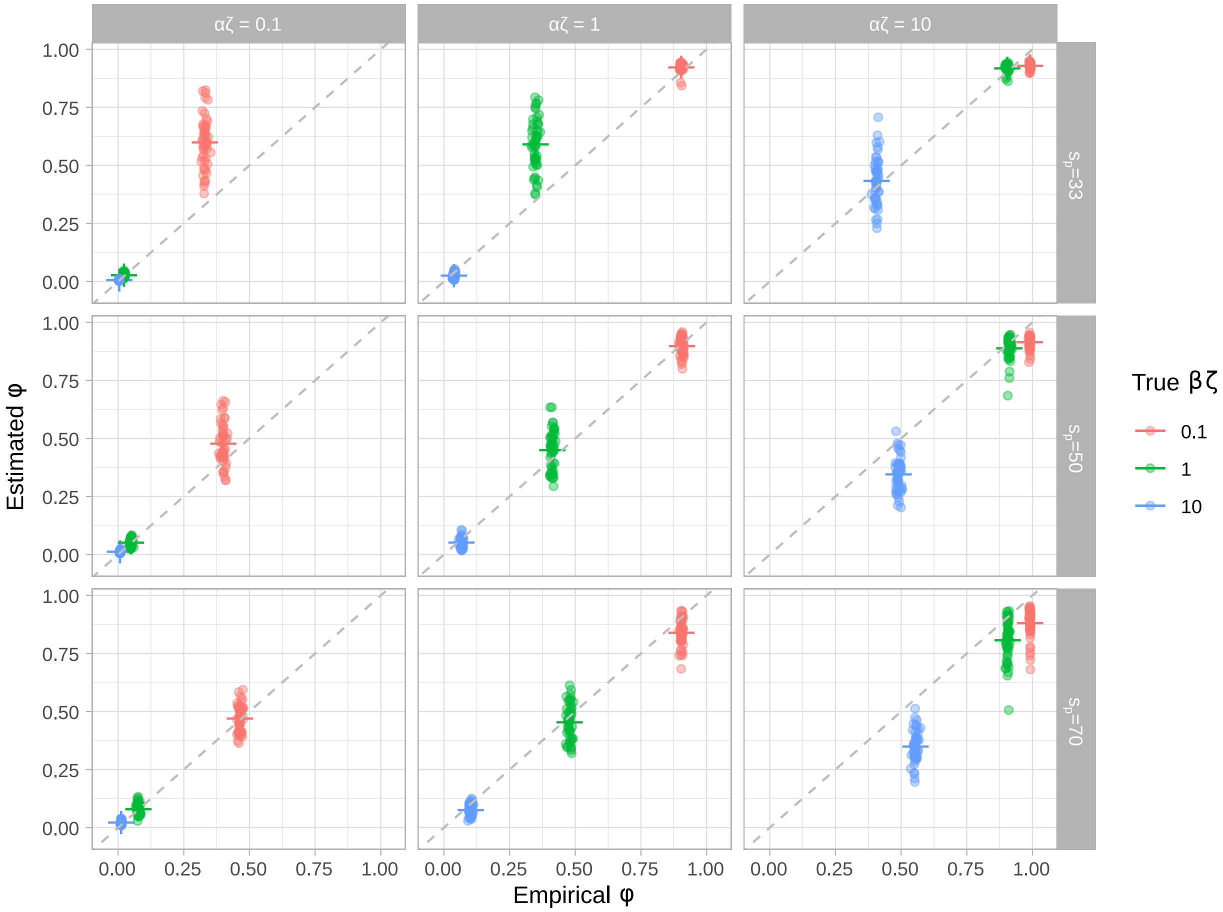

Supplementary Figure S10: Accuracy of estimated landing-pad occupancy ϕ under the steric hindrance model, for various values of $\alpha\zeta$ (columns), the spacing s_p between RNAPs (rows), and $\beta\zeta$ (see key). Scatter plots show the fraction of simulated cells for which the first 33, 50, or 70 nt (the "landing-pad") are occupied by an RNAP at steady state ("Empirical ϕ") vs. the fraction predicted to be occupied under the model ("Estimated ϕ") based on the simulated NRS data. 50 simulations were performed per parameter combination. Dashed line indicates $y = x$, and colored crosses represent the means of the corresponding points. A value of $\zeta = 2$ kb/min is assumed, so that $\alpha\zeta$ and $\beta\zeta$ are in events per minute.

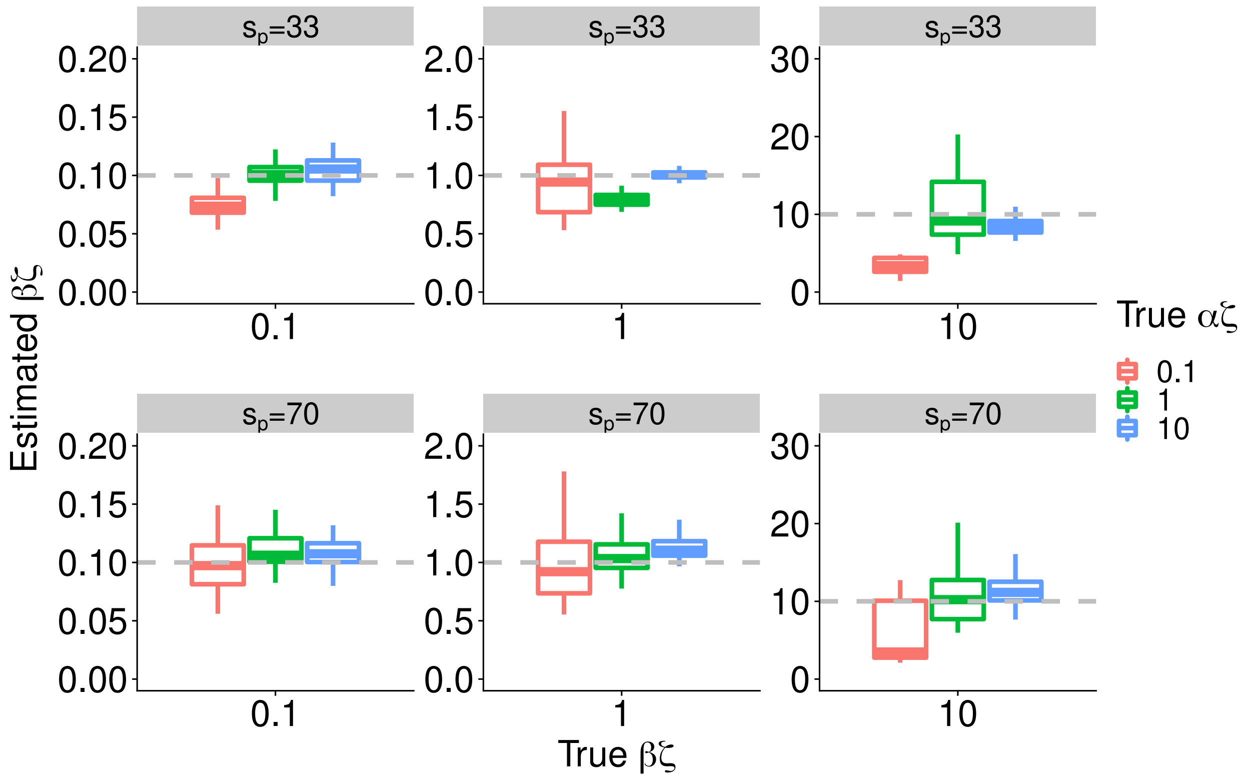

Supplementary Figure S11: Estimated values of $\beta\zeta$ under the steric hindrance model with alternative choices of the spacing parameter s_p (rows). Results are shown for simulated true values of $\beta\zeta \in \{0.1, 1.0, 10\}$ (left to right) and $\alpha\zeta \in \{0.1, 1, 10\}$ (see key). As in previous plots, dashed lines indicate the ground truth; boxplots summarize 50 replicates of the simulation; box boundaries indicate 1st and 3rd quartiles, and horizontal line indicates median. A value of $\zeta = 2$ kb/min is assumed so that $\alpha\zeta$ and $\beta\zeta$ can be assumed to have units of events per minute. Pause sites are variable with a mean position of $k = 50$ nt and a standard deviation of 25 nt.

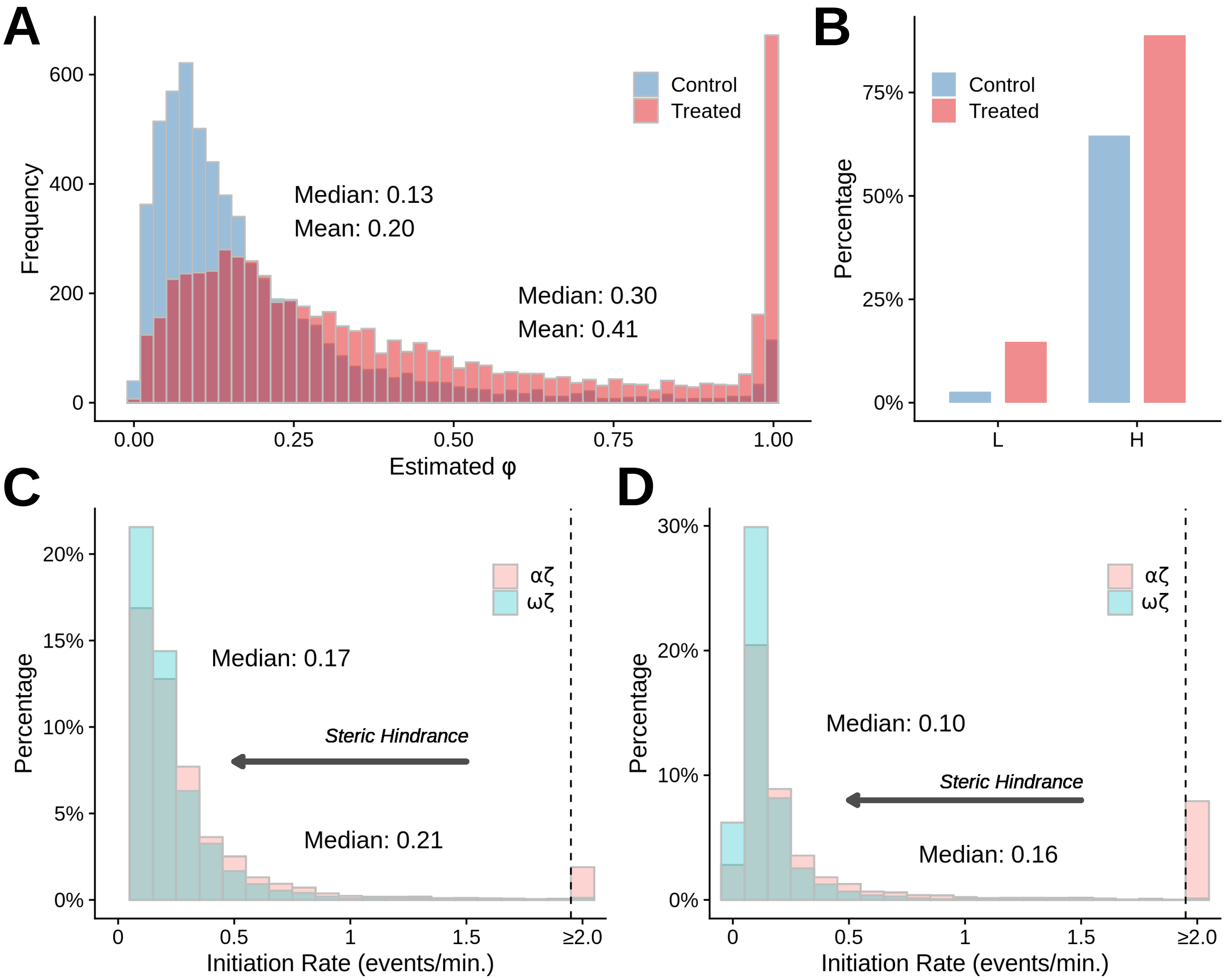

Supplementary Figure S12: **A.** Distribution of estimated ϕ for 5,964 robustly expressed genes in K562 cells before (Control) and after (Treated) treatment with celastrol under the low (L) calibration [2] (see **Materials & Methods** for details). **B.** Percentages of genes having fully occupied landing-pads ($\phi > 0.95$) before (Control) and after (Treated) treatment with celastrol, under the low (L) and high (H) calibrations. **C & D.** Distributions of scaled estimates of the "effective" ($\omega\zeta$) and "potential" ($\alpha\zeta$) rates of transcription initiation, in events per minute per cell, for the same genes. Panel **C** represents the NHS case and panel **D** represents the HS case. The x-axes are truncated to highlight the bulk of the distributions. Gray arrows indicate effects of steric hindrance.

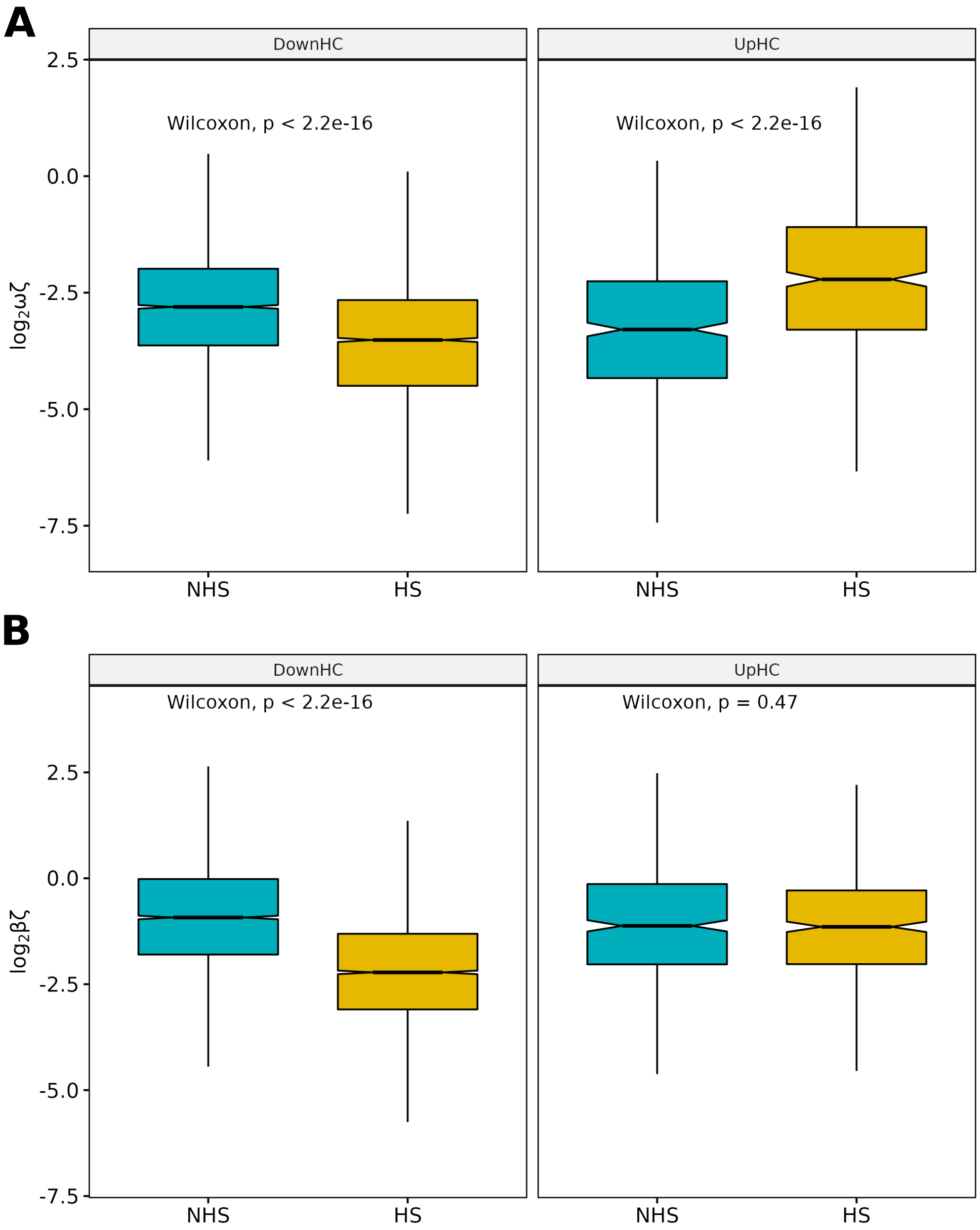

Supplementary Figure S13: **A.** Distribution of estimated $\omega\zeta$ for 4,399 heat-repressed (Down-regulated with High Confidence, DownHC) and 503 heat-induced genes (UpHC) in K562 cells. **B.** Distribution of estimated $\beta\zeta$ for the same group of heat-repressed or heat-induced genes. Gene sets were defined by Vihervaara et al. (2017) [1]. NHS and HS indicate $\omega\zeta$ or $\beta\zeta$ estimates before and after heat shock, respectively. $\omega\zeta$ was calibrated using the low (L) calibration as in **Fig. 5** (see also **Materials & Methods** for details).

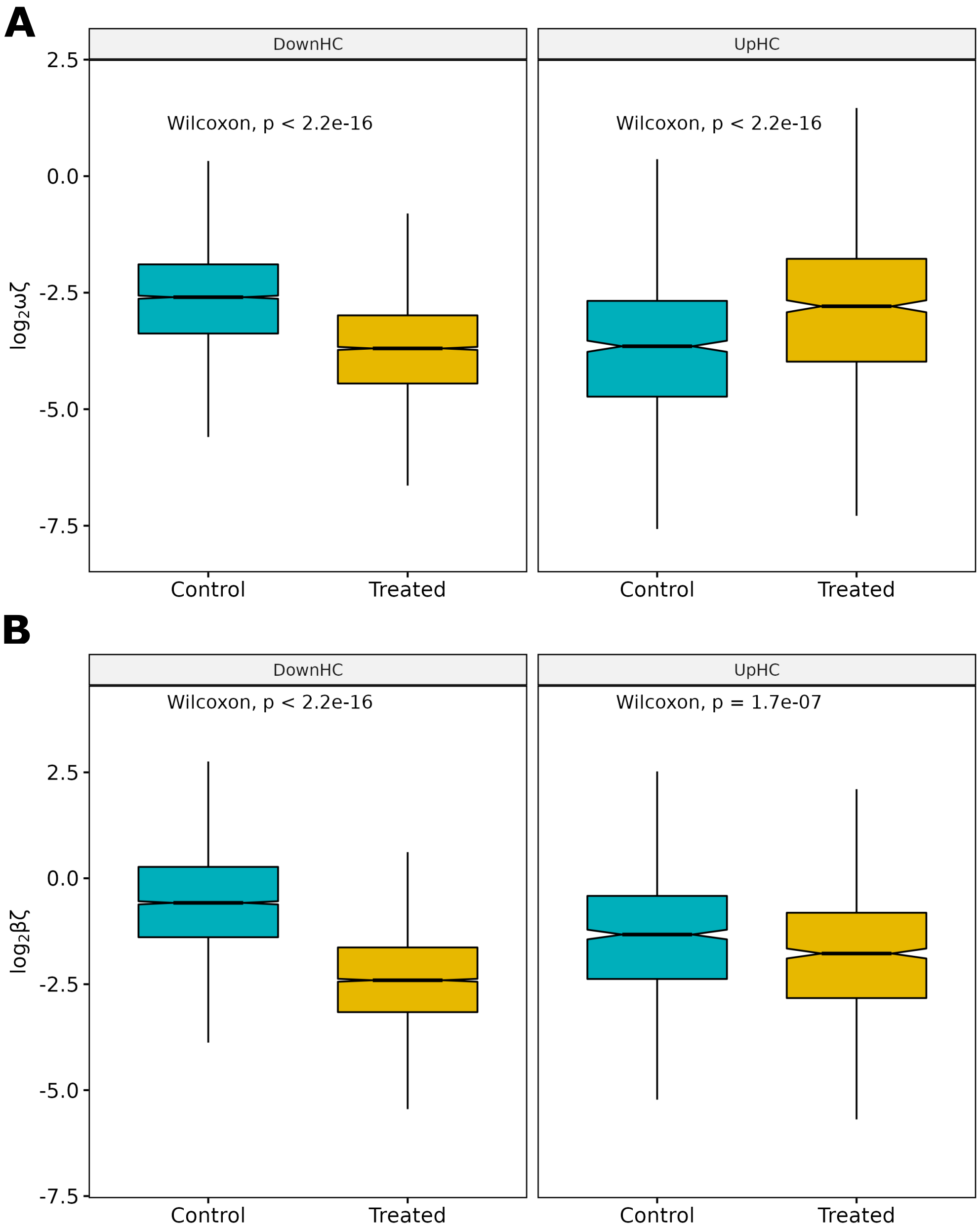

Supplementary Figure S14: **A.** Distribution of estimated $\omega\zeta$ for 4920 down-regulated (with High Confidence, DownHC) and 736 up-regulated genes (UpHC) in K562 cells after celastrol treatment [2]. **B.** Distribution of estimated $\beta\zeta$ for the same group of down-regulated or up-regulated genes. Control and treated samples indicate $\omega\zeta$ or $\beta\zeta$ estimates before and after celastrol treatment, respectively. $\omega\zeta$ was calibrated using the low (L) calibration as in **Fig. 5** (see also **Materials & Methods** for details).

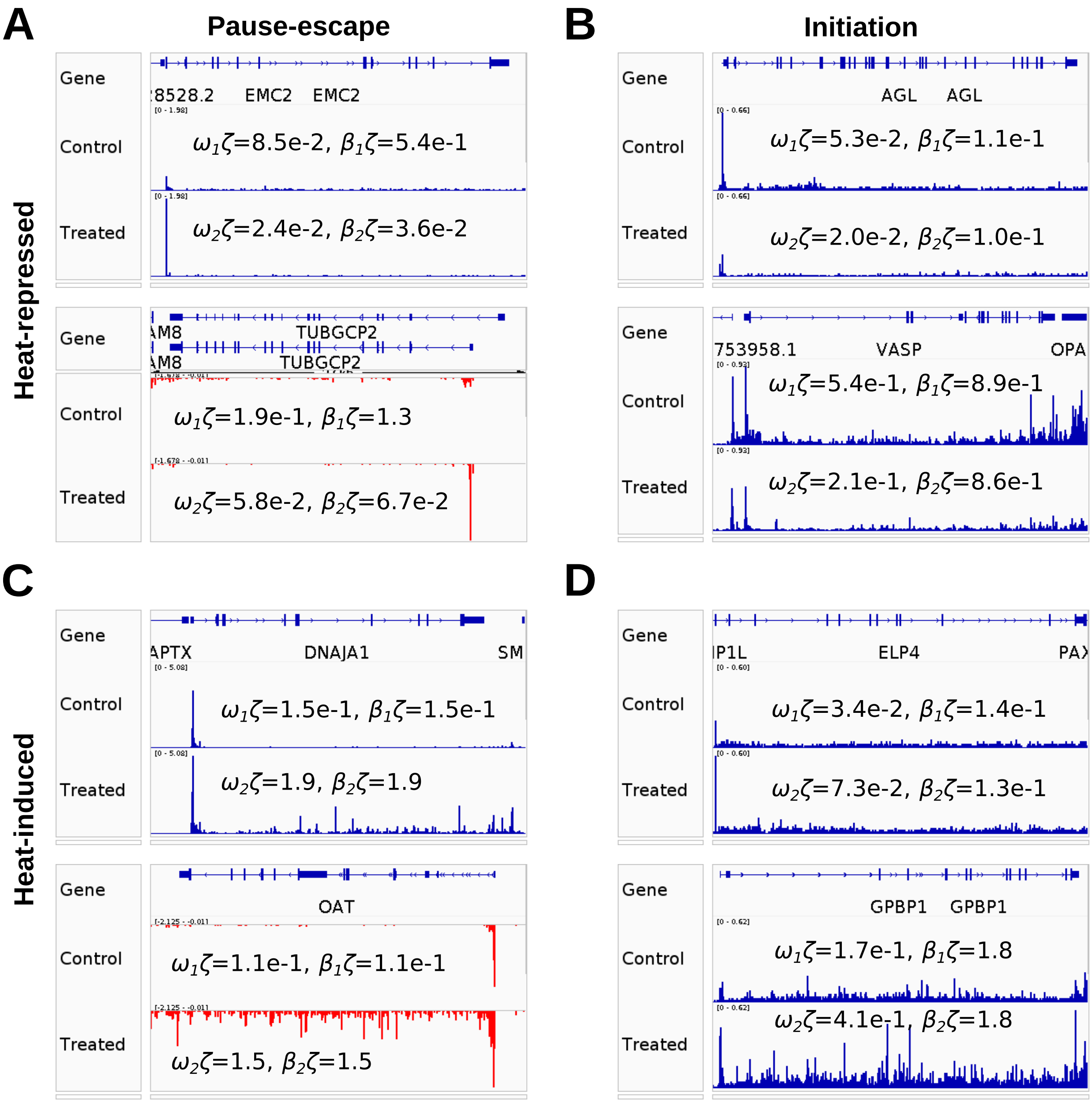

Supplementary Figure S15: PRO-seq data for selected heat-repressed (panels **A&B**) or heat-induced (panels **C&D**) genes apparently driven by changes in pause-escape rate (**A&C**) or initiation rate (**B&D**). $\omega\zeta$ was calibrated using the low (L) calibration as in **Fig. 5**. Rates were estimated before (Control) and after heat shock (Treated) [1]. PRO-seq signals were normalized in reads per million for visualization.

References

[1] Vihervaara, A. *et al.* Transcriptional response to stress is pre-wired by promoter and enhancer architecture. *Nat Commun* **8**, 255 (2017).

[2] Dukler, N. *et al.* Nascent RNA sequencing reveals a dynamic global transcriptional response at genes and enhancers to the natural medicinal compound celastrol. *Genome Res* **27**, 1816–1829 (2017).

[3] Bailey, T. L. STREME: Accurate and versatile sequence motif discovery. *Bioinformatics* (2021).

[4] Gupta, S., Stamatoyannopoulos, J. A., Bailey, T. L. & Noble, W. S. Quantifying similarity between motifs. *Genome Biol* **8**, R24 (2007).

[5] Dunham, I. *et al.* An integrated encyclopedia of DNA elements in the human genome. *Nature* **489**, 57–74 (2012).
